# Genome-Wide Expression Patterns and the Genetic Architecture of a Fundamental Social Trait

**DOI:** 10.1371/journal.pgen.1000127

**Published:** 2008-07-18

**Authors:** John Wang, Kenneth G. Ross, Laurent Keller

**Affiliations:** 1 Department of Ecology and Evolution, University of Lausanne, Lausanne, Switzerland; 2 Department of Entomology, University of Georgia, Athens, Georgia, United States of America; Princeton University, United States of America

## Abstract

Explaining how interactions between genes and the environment influence social behavior is a fundamental research goal, yet there is limited relevant information for species exhibiting natural variation in social organization. The fire ant *Solenopsis invicta* is characterized by a remarkable form of social polymorphism, with the presence of one or several queens per colony and the expression of other phenotypic and behavioral differences being completely associated with allelic variation at a single Mendelian factor marked by the gene *Gp-9*. Microarray analyses of adult workers revealed that differences in the *Gp-9* genotype are associated with the differential expression of an unexpectedly small number of genes, many of which have predicted functions, implying a role in chemical communication relevant to the regulation of colony queen number. Even more surprisingly, worker gene expression profiles are more strongly influenced by indirect effects associated with the *Gp-9* genotypic composition within their colony than by the direct effect of their own *Gp-9* genotype. This constitutes an unusual example of an “extended phenotype” and suggests a complex genetic architecture with a single Mendelian factor, directly and indirectly influencing the individual behaviors that, in aggregate, produce an emergent colony-level phenotype.

## Author Summary

Fundamental research goals for scientists interested in social evolution are to determine the numbers and types of genes that directly regulate individual social behaviors as well as to understand how the social environment indirectly influences the expression of socially relevant traits. The fire ant *Solenopsis invicta* features a remarkable form of social variation in which the occurrence of two distinct social types that differ in colony queen number is associated with genetic differences at a genomic region marked by the gene *Gp-9*. Our analyses of gene expression profiles in fire ant workers revealed that differences in *Gp-9* genotype are associated with the differential expression of an unexpectedly small number of genes, many of which are predicted to function in chemical communication relevant to the regulation of colony queen number. Surprisingly, worker gene expression profiles are more strongly influenced by indirect effects associated with the social environment within their colony than by the direct effect of their own *Gp-9* genotype. These results suggest a complex genetic architecture underlying the control of colony queen number in fire ants, with a single Mendelian factor directly regulating, and the social environment indirectly influencing, the expression of the individual behaviors that, in aggregate, yield an emergent colony social organization.

## Introduction

Considerable interest surrounds the genetic architectures underlying fundamental adaptive traits in wild populations [1]–[5]. In social organisms, such interest centers on the numbers and types of genes directly regulating expression of the individual behaviors that, in aggregate, create social organization, as well as genes in interactants that indirectly influence expression of socially relevant behaviors by altering the social environment [6]–[12]. This indirect influence is mediated by interactions of the genotype of a given individual with those of other group members who collectively comprise the social environment. Information on the genetic architecture of social organization is essential to constructing realistic models of social evolution that can answer questions about the numbers and types of genetic changes necessary to change a solitary to a social animal or to convert a simple society to a large and highly complex one [13].

A remarkable case of a fundamental social polymorphism that appears to be under simple genetic control (single Mendelian factor of large effect) is variation in colony social organization in the fire ant *Solenopsis invicta*. In this species a single genomic element marked by the protein-encoding gene *Gp-9* is implicated in the production of two distinct types of queens that differ in physiology, fecundity and behavior [14]–[19]. This genetic factor also determines whether workers tolerate a single fertile queen (monogyne social form) or multiple queens (polygyne social form) in their colony. Colonies containing only homozygous *Gp-9 BB* workers accept only a single queen, whereas colonies containing both *Gp-9 BB* and *Gp-9 Bb* workers invariably accept multiple queens, but only those bearing a *Gp-9 b* haplotype [20]–[23]. The near complete absence of adult workers and queens with a *bb* genotype stems from the deleterious effects associated with the genomic region marked by the *b* allele, inducing homozygous females to die shortly after they eclose from the pupa [22],[24]. The monogyne and polygyne social forms also differ in a number of important reproductive, behavioral, and life history traits besides colony queen number [25],[26], differences that are also completely associated with differences at the genomic region marked by *Gp-9*. In contrast, there is a complete lack of differentiation at genes not tightly linked to *Gp-9*, presumably because frequent matings between sexuals from sympatric monogyne and polygyne colonies result in extensive gene flow between the forms [22], [27]–[29].

Colony queen number in *S. invicta* is regulated by the workers, which collectively decide which and how many queens from within or outside the colony are recruited as new egg-layers [21], largely on the basis of chemical signals emanating from the queens [20]. Workers in monogyne colonies (all of which possess the *BB* genotype) accept only a single replacement queen that must also bear genotype *BB*, whereas workers in polygyne colonies accept multiple queens, each of which must possess the *b* haplotype. Significantly, the presence of as few as 5–10% of workers with the *b* haplotype induces the entire colony worker force, including *BB* workers, to become tolerant of multiple *Bb* queens and thus display the polygyne social phenotype [23]. Thus the genomic region marked by *Gp-9* exerts indirect genetic effects [9], in that the presence of the *b* variant in a colony induces changes in the social behavior of all colony members, even those lacking the *b* haplotype. Although the identity of the product of *Gp-9* as an odorant binding protein (OBP) and other lines of evidence suggest that the gene may play a direct part in regulating social organization via a role in chemical communication, it remains an open question whether other genes tightly linked to *Gp-9* (possibly locked up in an inversion with it) are also involved [22]. The first aim of this study was to investigate whether variation in the genomic region marked by *Gp-9* is associated with differences in patterns of expression of genes other than *Gp-9* in workers. The second aim was to study how the social environment (i.e., presence or absence of nestmate workers with the *b* allele) can alter individual gene expression patterns. To answer these questions and begin to address the issue of how variation at a single Mendelian factor can directly and indirectly affect gene expression to produce a complex colony-level phenotype, we employed a fire ant microarray platform representing some 10,000 genes [30].

## Results/Discussion

**Figure 1:**
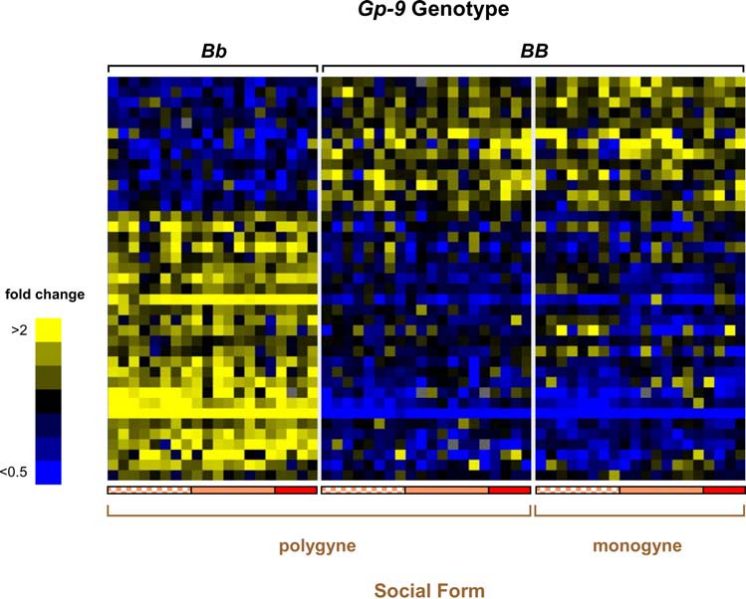
Expression profiles between *S. invicta* adult workers with the *BB* and *Bb* genotypes of *Gp-9*. Expression profiles for 39 differentially expressed genes are depicted (ANOVA, 10% false discovery rate [FDR]). Each row represents data for one gene, and each column represents data for a pool of 7–10 nestmates with the same *Gp-9* genotype sampled from each of twenty colonies of each social form. Colonies were collected from Georgia (2004, checkered peach bar; 2006, solid peach bar) and Louisiana (2006, solid red bar), USA (data from each polygyne colony are presented in the identical order for the alternate genotype groupings). Expression levels for each gene are depicted relative to the average level across all experimental samples (blue, low levels; yellow, high levels). Genes are arranged by hierarchical clustering. See Text S1 and Table S1 for confirmation of selected gene expression results with quantitative RT-PCR (qRT-PCR).

To determine the effect of *Gp-9* genotype on gene expression in focal individuals, we compared expression profiles between *BB* and *Bb* adult workers from 20 polygyne *S. invicta* colonies. This comparison revealed 39 genes consistently differentially expressed between workers of the two genotypes, of which about two-thirds were more highly expressed in *Bb* than *BB* workers (Figure 1; see also confirmation of microarray data by quantitative RT-PCR [qRT-PCR] in Text S1 and Table S1). Sixteen of these genes did not significantly match any sequence in the public databases (BLASTX, threshold *E*-value = 1e-5), ten matched predicted proteins of unknown function, and the remaining 13 matched genes with a known or inferred function (Table 1).

**Table 1:** Genes differentially expressed between polygyne *S. invicta* workers bearing *Gp-9* genotypes *Bb* and *BB* that significantly match annotated genes in public databases^a^.

Fire ant gene	Putative gene product of best match^b^	*E*-value	Gene category	Expression Ratio (*Bb/BB*)^c^	*P*-value^d^
SI.CL.18.cl.1888.Contig1^e^	odorant binding protein homolog #1 (Q5EP09)	5.00E-10	odorant binding	0.49	1.17E-04
SI.CL.40.cl.4070.Contig1	venom allergen homolog (P35778)	6.00E-72	allergen	0.53	2.88E-04
SI.CL.1.cl.162.Contig1^e^	antennae-specific chemosensory protein homolog (Q2VW29)	3.00E-17	odorant binding	0.54	6.71E-06
SiJWG04ABQ.scf	*S. invicta* venom allergen 3 (P35778)	1.00E-136	allergen	0.55	1.21E-05
SI.CL.5.cl.547.Contig1	Step ii splicing factor slu7 (Q16FY9)	1.00E-85	RNA processing	0.63	4.95E-06
SI.CL.24.cl.2429.Contig1	Proteasome subunit alpha type 2 (Q8T0Y8)	1.00E-114	protein degradation	0.77	2.62E-06
SI.CL.0.cl.015.Contig2^e^	mitochondrial Ribosomal protein L21 (Q29DI1)	6.00E-36	mitochondrial	***1.41***	7.27E-05
SiJWH01ABW.scf	low density lipoprotein receptor-related protein associated protein (lrpap1) (A2I465)	2.00E-34	receptor modulation	***1.57***	1.15E-11
SI.CL.3.cl.385.Contig1	odorant binding protein homolog #2 (Q8WRP9)	2.00E-06	odorant binding	***1.69***	4.69E-05
SiJWC05ACO.scf	growth-arrest-specific protein 8 (ENSCSAVP00000007111)	2.00E-51	cytoskeleton regulation	***2.18***	4.33E-11
SiJWC03CAW.scf	TatD related deoxyribonuclease (ENSCINP00000014669)	9.00E-09	nucleic acid metabolism	***2.44***	4.84E-09
SI.CL.26.cl.2690.Contig1	BEL-PAO transposon polyprotein (Q4JS97)	4.00E-32	transposon	***3.61***	5.41E-15
SiJWF04BEA.scf	piggyBac transposon (Q75R41)	6.00E-15	transposon	***28.94***	3.10E-27

a Threshold, *E*<1e-5. See Table S3 for all BLASTX matches with *E*≤1 for all 39 differentially expressed genes.

b Excludes Ensembl *Apis* gene predictions. Accession numbers of best matches (TrEMBL, Swiss-Prot, or Ensembl databases) are shown in parentheses.

c Expression ratios are based on averages for all *Bb* and *BB* workers in the 20 polygyne study colonies. Elevated expression in *Bb* workers relative to *BB* workers is highlighted with bold italics.

d *P*-values from ANOVA calculations are averages for genes represented by more than one significantly differentially expressed clone on the microarray (10% FDR).

e Assembled sequence is composite of separate contigs that were merged because they have >95% sequence identity.

Three gene categories were overrepresented among the genes differently expressed between workers of alternate genotypes (Table 1 and Table S2; all *P*<0.05). The first two are the allergen and odorant binding protein categories, which collectively include five genes likely to contribute to chemical signaling and response, the essential components of regulation of colony queen number and social organization. In ants, venom allergens are proteins released from the venom sac, an organ that, in queens, appears to store and release chemical signals allowing recognition by workers [25]. Similarly, the two odorant binding genes that encode members of the insect OBP protein family (as does *Gp-9*) and the antennal chemosensory protein may be involved in pheromone transduction, thereby potentially influencing the abilities of workers of the two *Gp-9* genotypes to recognize and discriminate among queens. Experiments from other systems are suggestive that changes in the expression levels of OBPs could influence discriminatory behavior by modulating the threshold for a particular response; differential regulation of OBPs has been observed in *Drosophila* following mating [31], exposure to starvation stress [32], and alcohol tolerance development after exposure to alcohol [33]. Additionally, genetic and biochemical evidence suggests that OBPs may interact combinatorially in odor discrimination [34],[35].

The third overrepresented category comprises two transposons, which are of special interest with respect to properties that may be shared between the genomic region including *Gp-9* and regions containing the sex-determining genes in species with sex chromosomes [36]. The *b* haplotype is found only in the polygyne social form, just as the Y chromosome is found only in males in species with male heterogamety. By analogy with the Y chromosome, theoretical predictions and empirical observations suggest that the *Gp-9 b* region should (i) accumulate genes beneficial in the polygyne social environment (as the Y chromosome accumulates genes beneficial to male function [37]), (ii) evolve reduced recombination to preserve associations of genes advantageous for polygyny (as occurs for genes advantageous to males on the Y chromosome [38],[39]), and (iii) accumulate deleterious alleles and transposable elements (because of reduced recombination [38],[39]). Consistent with these expectations, the genomic region marked by *Gp-9* is characterized by low recombination [40],[41], the *b* haplotype is a homozygous lethal [15],[24],[40], and the *piggyBac*-like transposon, which is differentially expressed between workers of alternate genotypes, appears to occur almost exclusively in individuals possessing haplotype *b* (data not shown). Thus the strong expression of at least this transposon in *b*-bearing workers, which constitutes the most extreme expression difference among the 13 genes with annotated matches (Table 1), likely reflects its unique insertion in *b* haplotypes. While this distribution could signify that the *piggyBac*-like transposon directly affects the differential expression of other candidate genes in *BB* and *Bb* workers, we note that, consistent with earlier protein electrophoresis data [40], no significant difference in the expression levels of *Gp-9* was detected between workers of the two genotypes; therefore, whatever elements control the differential expression of genes in parallel with *Gp-9* genotype appear not to regulate the expression of *Gp-9* itself.

**Figure 2:**
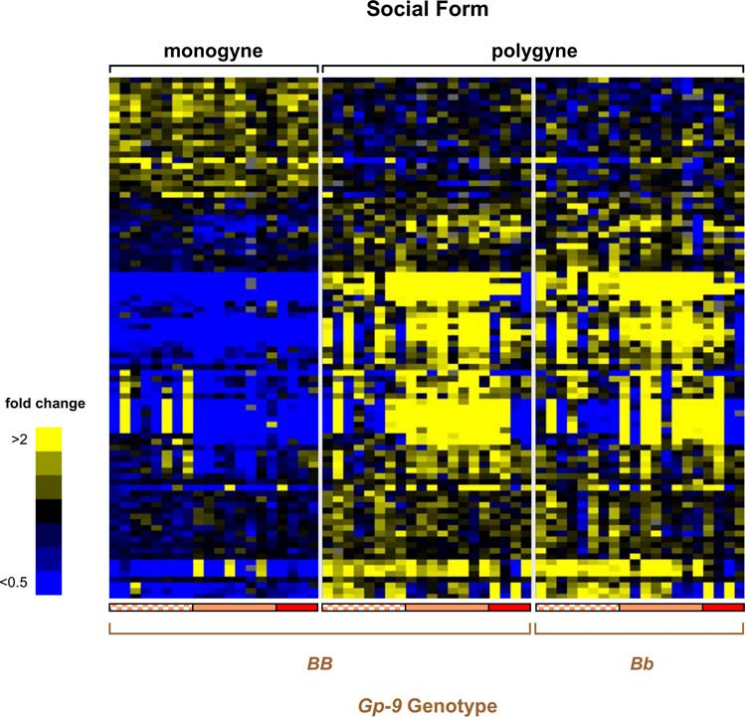
Expression profiles between *S. invicta* adult workers from monogyne and polygyne colonies. Expression profiles for 91 differentially expressed genes are depicted (ANOVA, 10% FDR). Rows and columns represent data as described in the Figure 1 caption. See Text S1 and Table S1 for confirmation of selected gene expression results with qRT-PCR.

To determine the indirect effects of colony *Gp-9* genotype composition as well as other aspects of the social environment on worker gene expression, while controlling for individual *Gp-9* genotype, we compared profiles of adult workers bearing genotype *BB* between 20 polygyne and 20 monogyne colonies. This comparison revealed 91 genes consistently differentially expressed between workers of the alternate forms, of which over three-quarters were more highly expressed in polygyne than monogyne workers (Figure 2; see also confirmation of microarray data by qRT-PCR in Text S1 and Table S1). Forty-five of these genes did not significantly match any sequence in the public databases (BLASTX, threshold *E*-value = 1e-5), 13 matched predicted proteins of unknown function, and the remaining 33 matched previously annotated genes (Table 2).

**Table 2:** Genes differentially expressed between *S. invicta* workers from monogyne and polygyne colonies bearing the *BB* genotype at *Gp-9* that significantly match annotated genes in public databases^a^.

Fire ant gene	Putative gene product of best match^b^	*E*-value	Gene category	Expression Ratio (P/M)^c^	*P*-value^d^
SiJWH11BCZ2.scf	Y-box protein (A2A246)	1.00E-38	regulation of transcription	0.69	7.24E-07
SI.CL.20.cl.2059.Contig1	defensin-2 (Q5MQL3)	3.00E-15	immunity	0.70	8.39E-05
SI.CL.2.cl.203.Contig1	prenylcysteine oxidase (ENSDARP00000029873)	1.00E-21	oxidoreductase	0.77	3.98E-04
SI.CL.11.cl.1163.Contig1	alpha-glucosidase (hbg3) (Q25BT6)	8.00E-55	metabolism	0.78	4.39E-04
SI.CL.31.cl.3193.Contig1	S-methyl-5-thioadenosine phosphorylase (AGAP005129-PA)	1.00E-44	transferase	0.79	4.72E-05
SiJWF02ABX.scf	alanine-glyoxylate aminotransferase (Q6DIW8)	3.00E-17	transferase	0.84	7.20E-05
SI.CL.5.cl.571.Contig1	ribosomal protein L3 (Q56FI0)	0	translation	0.84	3.32E-04
SI.CL.6.cl.615.Contig1	probable allergen protein (O18530)	5.00E-27	allergen	0.89	2.34E-04
SI.CL.30.cl.3064.Contig1	mitochondrial import inner membrane translocase subunit Tim9 (Q17HY2)	1.00E-28	mitochondrial	***1.14***	7.25E-04
SI.CL.11.cl.1166.Contig2	dynein light chain 2B (Q17AA0)	6.00E-43	microtubule motor activity	***1.15***	8.58E-05
SI.CL.21.cl.2171.Contig1	prefoldin subunit 4 (Q17IJ1)	1.00E-36	prefoldin chaperone	***1.17***	2.84E-04
SI.CL.25.cl.2556.Contig2	prefoldin subunit 6 (A2I449)	2.00E-30	prefoldin chaperone	***1.17***	7.62E-04
SI.CL.0.cl.000.Contig1^e^	prefoldin subunit 2 (Q16LV2)	7.00E-11	prefoldin chaperone	***1.18***	7.32E-04
SI.CL.1.cl.105.Contig3	c-Myc-binding protein (Q8R048)	6.00E-12	regulation of MYC	***1.24***	7.47E-04
SiJWE04AAB.scf	mitochondrial ribosomal protein L32 (Q29BC5)	4.00E-29	mitochondrial	***1.25***	1.01E-04
SI.CL.12.cl.1258.Contig1	succinate-ubiquinone reductase membrane anchor subunit, mitochondrial (Q9VCI5)	3.00E-28	mitochondrial	***1.28***	3.24E-04
SI.CL.10.cl.1028.Contig1	small nuclear ribonucleoprotein G (snRNP-G) (Q56FI6)	1.00E-32	RNA processing	***1.29***	4.57E-04
SI.CL.3.cl.341.Contig1	mitochondrial import receptor subunit TOM7 (Q4TC18)	1.00E-11	mitochondrial	***1.29***	9.18E-05
SI.CL.7.cl.737.Contig1	putative Deoxycytidylate deaminase (Q16GS2)	7.00E-72	nucleic acid metabolism	***1.29***	4.87E-04
SI.CL.5.cl.551.SiJWC10BDQ.scf	ATP synthase-like protein, mitochondrial (Q0PXW9)	5.00E-07	mitochondrial	***1.41***	2.12E-04
SI.CL.40.cl.4000.Contig1	DNA polymerase v (Q17DX3)	1.00E-09	DNA replication	***1.51***	8.54E-06
SI.CL.9.cl.997.Contig1	septin-2 (Q29BR7)	1.00E-173	cell division	***1.55***	2.00E-05
SI.CL.9.cl.942.Contig1^e^	ribonuclease H (Q5AC61)	1.00E-11	nucleic acid metabolism	***1.74***	2.11E-04
SI.CL.4.cl.464.SiJWC10BAB.scf	cytochrome b (cytB) (Q6RVT3)	2.00E-59	mitochondrial	***2.14***	5.13E-05
SI.CL.0.cl.041.SiJWH03BDX.scf	cytochrome oxidase subunit I (COI) (Q1PLX5)	1.00E-73	mitochondrial	***2.21***	9.20E-05
SI.CL.31.cl.3197.Contig1	ribosomal protein L22, mitochondrial (Q29IK4)	2.00E-55	mitochondrial	***2.81***	1.06E-05
SI.CL.14.cl.1449.Contig1	bone morphogenetic protein (Q16JR6)	9.00E-51	intercellular signaling	***2.92***	2.27E-04
SI.CL.41.cl.4135.Contig1	polyprotein [ssRNA(+) virus] (Q38QJ4)	4.00E-15	virus	***3.87***	4.71E-04
SI.CL.18.cl.1832.Contig1	putative structural protein of *S. invicta* virus 2 (A5HB91)	2.00E-81	virus	***5.90***	2.88E-06
SI.CL.28.cl.2823.Contig1	polyprotein [ssRNA(+) virus] (Q38QJ4)	1.00E-33	virus	***12.57***	1.12E-04
SI.CL.42.cl.4295.Contig1	putative structural protein of *S. invicta* virus 2 (A5HB89)	1.00E-120	virus	***14.30***	3.75E-09
SI.CL.25.cl.2511.Contig1	(pv4)Non-capsid protein [ssRNA(−) virus] (O11437)	9.00E-11	virus	***51.98***	1.14E-11
SI.CL.6.cl.610.Contig1^e^	non-structural protein of *S. invicta* virus 2 (A5HB92)	1.00E-126	virus	***87.39***	6.73E-10

a Threshold, *E*<1e-5. See Table S4 for all BLASTX matches with *E*≤1 for all 91 differentially expressed genes.

b Excludes Ensembl *Apis* gene predictions. Accession numbers of best matches (TrEMBL, Swiss-Prot, or Ensembl databases) are shown in parentheses.

c Expression ratios are based on averages for all monogyne (M) and polygyne (P) *BB* workers in the 40 study colonies. Elevated expression in P workers relative to M workers is highlighted with bold italics.

d *P*-values from ANOVA calculations are averages for genes represented by more than one significantly differentially expressed clone on the microarray (10% FDR).

e Assembled sequence is composite of separate contigs that were merged because they have >95% sequence identity.

Three gene categories (mitochondrial, prefoldin complex, and viral genes) were overrepresented among the genes that were differentially expressed between *BB* workers from monogyne and polygyne colonies (Table 2 and Table S2; all *P*<0.05). The 11 genes encoding mitochondrial and prefoldin complex (molecular chaperone) proteins were all up-regulated in polygyne compared to monogyne workers. Increased mitochondrial gene expression may reflect increased oxidative metabolism, while increased prefoldin expression may indicate higher protein synthesis rates, possibly in relation to the relatively smaller size and higher metabolic rates of polygyne workers [42].

The pattern of expression of the six genes in the viral gene category is consistent with the expectation that differences in social organization affect susceptibility to pathogens and parasites. In the monogyne form, there is intense selection against susceptible infected individuals during independent colony founding, a stage that colonies of the polygyne form never display [26]. Accordingly, we found that workers in the polygyne form express more sequences corresponding to viral genes than their counterparts in the monogyne form, presumably because of relaxed selection and generally greater susceptibility in the former (see also [43]). Based on sequence similarity and correlated expression across our experiments, we identified six gene products that likely represent three different viruses, a ssRNA negative-strand (−) virus and two ssRNA positive-strand (+) viruses, one of which is the SINV-2 virus [44]. All 20 polygyne study colonies showed evidence of infection with at least one virus (mean number of viral types per colony, 2.5±0.67), whereas only three of the 20 monogyne colonies showed evidence of infection, in all cases by a single viral type. Finally, the pattern of expression of another socially-regulated gene, this one encoding a defensin (a class of small protein antibiotics active against viruses, bacteria, and fungi [45]), also is consistent with greater selection for resistance in the monogyne form, as this gene was more highly expressed in monogyne than polygyne workers.

The numbers of genes differentially expressed in the genotype (39) and social form (91) comparisons are relatively low compared to the numbers expected based on other published microarray experiments. There are several possible explanations for this. First, the use of whole worker bodies as the source of RNA may decrease the probability of detecting genes whose level of expression varies among cells or tissues. Second, our comparisons were performed on groups of workers originating from different colonies, thus adding a colony-level effect to, and thus increasing the total variance in, gene expression. Finally, workers of alternate genotype or social form apparently exhibit fewer phenotypic differences than queens [20],[21],[46],[47], possibly reflecting the involvement of fewer differentially expressed genes in the former caste.

Remarkably, there was almost no overlap between genes whose level of expression was influenced by the focal workers' *Gp-9* genotypes and genes whose expression was influenced by the social environment, with only one of the 129 differentially expressed genes appearing in both categories. This demonstrates an almost complete decoupling of the direct effects of the genomic region marked by *Gp-9* and the indirect effects mediated by social interactions within colonies. Moreover, there is little indication of an interaction between these direct and indirect effects; genes whose levels of expression depend on the *Gp-9* genotype of focal individuals generally are expressed at similar levels in the two social forms when genotype is held constant (Figure 1), whereas genes whose levels of expression depend on the indirect influence of the social environment almost always are expressed at similar levels in polygyne workers of different *Gp-9* genotypes (Figure 2; see also Figure S1).

This study reveals that variation at the *S. invicta* genomic region marked by *Gp-9* is associated with the differential expression in workers of a relatively small number of genes that, with the exception of the *piggyBac*-like transposon, presumably are unlinked to *Gp-9*. A high proportion of these differentially expressed genes have putative functions implying a role in chemical signaling and behavior relevant to the regulation of colony queen number and, therefore, these genes may have a primary function in determining social organization. The number of such genes is unexpectedly low given the profound behavioral, physiological, and life-history differences between the two social forms and the fact that widespread changes in gene expression patterns can be observed after just a few generations of selection [48],[49]. A perhaps more surprising finding is that worker gene expression profiles are significantly more strongly influenced by indirect effects associated with the *Gp-9* genotypic composition within their colony than by the direct effect of their own *Gp-9* genotype (chi-squared test, *P*<0.001), with the indirect-effect genes largely implicated in the secondary differences in colony social characteristics expected between the forms. While several studies have demonstrated that the social environment can modulate gene expression [50]–[53], and others have revealed indirect genetic effects on phenotypes or levels of gene expression [10], [54]–[60], this is the first example of a naturally occurring polymorphic Mendelian element that affects gene expression in other group members. The finding of a complex genetic architecture directly and indirectly influencing the individual behaviors that, in aggregate, generate a fundamental colony-level social phenotype represents an unusual example of an “extended phenotype” [61].

## Materials and Methods

### Sample Collection

Colonies of *S. invicta* were collected near Athens, Georgia (eight polygyne and eight monogyne colonies in 2004; eight polygyne and eight monogyne colonies in 2006) and near Hammond, Louisiana (four polygyne and four monogyne colonies in 2006), USA. All colonies were returned to the laboratory and reared for one month under standard conditions [62]. We determined the social form of each study colony using several lines of evidence. Nest density, worker size distribution, and nest brood composition were used to make initial identifications of social form in the field (see [63]). Subsequently, polygyny was confirmed for each suspected polygyne colony by discovering two or more wingless inseminated (reproductive) queens, while monogyny was confirmed in each suspected monogyne colony by discovering a single, highly physogastric, wingless inseminated queen. The social form of each colony was further substantiated by electrophoretically detecting the *b* allele of *Gp-9* in pooled samples of 20 female inhabitants of each polygyne colony and failing to detect the allele in such samples from each monogyne colony (the *b* allele is completely diagnostic for polygyny in *S. invicta* in the USA [14],[40],[63]).

### mRNA Isolation and Microarray Hybridization

From each polygyne colony, 24–40 medium-sized adult workers were haphazardly collected from the foraging area of each colony and individually flash-frozen with liquid nitrogen in tubes containing 1 g of 1.4 mm ceramic beads (Quackenbush). From each monogyne colony, 10 medium-sized adult workers were collected in an identical fashion. For the 2004 samples, individual ants were homogenized in Trizol (Invitrogen) using a Fastprep bead shaker, and DNA and RNA were extracted using the manufacturer's recommended protocol. For the 2006 samples, individual ants were homogenized in RLT+ buffer, and DNA and RNA were extracted using the AllPrep RNA/DNA Mini Kit (Qiagen). An RFLP analysis was used to determine the *Gp-9* genotype of each individual in polygyne colonies [14]. We pooled the RNA from 7–10 *BB* workers and 7–10 *Bb* workers from each polygyne colony. Although *bb* workers generally are rare due to deleterious effects associated with the genotype [24],[40], we found 13 such workers from eight colonies. Two pooled *bb* samples were created, one for 2004 (nine workers from four colonies) and one for 2006 (four workers from four colonies). These samples were hybridized (after amplification) but not included in the statistical analysis due to the small number and pooling of individuals across colonies. RNA from ten workers from each monogyne colony (all with genotype *BB*) was pooled by colony.

For both the 2004 and 2006 samples, pooled total RNA was linearly amplified once (Ambion MessageAmp II kit), then labeled using a modified version of the aminoallyl-labeling method in which reverse transcription is performed in the presence of aminoallyl-dUTP and the resulting cDNA is coupled to Cy3 or Cy5 fluorescent monomers [64],[65]. Briefly, amplified RNA (∼5 µg) was mixed with random 9mers (2 µg/ul), 0.5 µl of Alien mRNA Spike mix (Stratagene), and water for a final volume of 17.5 µL. This RNA/primer mix was incubated for 10 min at 70°C, then held for 5 min on ice. Reverse transcription was performed for 2 h at 50°C after adding 6 µL of 5× first-strand buffer, 3 µL of 0.1 M DTT, 0.6 µL of 50× aminoallyl-dNTP mix (25 mM dATP, 25 mM dCTP, 25 mM dGTP, 15 mM dTTP, 10 mM aminoallyl-dUTP), 1 µL of RNAse inhibitor (15 U/µL, Invitrogen), and 2 µL of SuperScript III reverse transcriptase (200 U/µL, Invitrogen). The RNA was then hydrolyzed by adding 15 µL of 0.1 M NaOH and incubating for 10 min at 70°C. The pH was neutralized by adding 15 µL of 0.1 M HCl.

The aminoallyl-labeled cDNA was purified with a modified Qiaquick PCR Purification Kit (QIAGEN) and coupled to Cy3 or Cy5 dyes [64]. The combined Cy3- and Cy5-labeled probes were purified using the Qiaquick PCR Purification Kit (QIAGEN) and eluted in 72 µL of elution buffer. After adding 13.5 µL of 20× SSC, 2.7 µL of yeast tRNA (2 µg/µL), 2.7 µL of polyA DNA (2µg/µL; Sigma), and 1.62 µL of 10% SDS, the probe was denatured at 100°C for 45 sec and hybridized to the microarray slides at 64°C overnight. Excess probe was removed by washing for 2×5 min in 2× SSC, 0.1% SDS; 2×1 min in 0.2× SSC; 1×1 min in 0.1× SSC; and 1×5 min in 0.1× SSC, 0.1% Triton at room temperature.

Experimental samples were labeled with Cy3 and were hybridized against Cy5-labeled “common reference” RNA on our custom-made spotted cDNA microarrays. We employed a common reference design because not all samples provided enough amplified RNA for multiple hybridizations (e.g., for loop designs) and because this allowed within-form comparisons of polygyne genotypes and between-form comparisons of *BB* workers. All experimental samples were labeled in Cy3, allowing for unbiased comparisons. We used two different batches of reference RNA. For the 2004 samples, we pooled 25% of the amplified RNA from each experimental sample. For the 2006 samples, we amplified total RNA isolated en masse from hundreds of adult workers collected from the foraging area of 30 colonies (eight and seven of each social form from Georgia and Louisiana, respectively). The microarrays were made from 22,560 independent cDNAs generated from a fire ant expressed sequence tag project and are estimated to represent 11,864 different genes [30]. Two different batches of microarrays were used, one set printed in 2004 and the other in 2006. For both batches, only the 18,438 spots yielding a single PCR product (representing 9,722 putative genes) were considered in the analyses. Images of the competitive hybridization were obtained with an Agilent Technologies Scanner. The signal intensities for each spot were extracted from the images using GenePix software. After scanning, bad spots were flagged and the background-subtracted median foreground values were used as the intensity levels in the subsequent analysis. All spots with a positive intensity were considered for the subsequent analyses (i.e., no threshold filtering was used). Raw intensity data were converted to normalized log2 ratios using “print-tip specific” loess normalization (within arrays; marray Bioconductor package, R [66]).

Selected gene expression results were confirmed using qRT-PCR (see Text S1 and Table S1). Primers used for qRT-PCR are listed in Table S5.

### Data Analysis

For the genotypic analysis, we tested for differential expression of each gene between samples of *BB* and *Bb* workers in the 20 polygyne colonies using a 2-factor mixed-model ANOVA of the form:
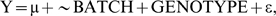
where Y, representing the reference/sample log-transformed ratio for a spot, is the sum of effects. The symbol μ represents the overall average log-transformed ratio for a given spot over all experiments. BATCH is a random effect (denoted by ∼) with two levels, batch_2004 and batch_2006, that accounts for the variation between hybridizations performed in the two different years (this “year” effect also encapsulates the effects of two different batches of microarrays and of distinct reference RNAs). The term GENOTYPE captures the gene expression changes that are attributable to the *BB* and *Bb* genotypes. Finally, ε represents the measurement error. We did not include data for the *bb* workers in the statistical analysis due to the small number of samples. However, these samples yielded expression profiles that appeared similar to those of *Bb* workers (but with even more marked differences from the profiles of *BB* workers, data not shown).

For the social form analysis, we tested for differential expression of each gene between *BB* samples from 20 monogyne and 20 polygyne colonies by using the same 2-factor mixed-model ANOVA, but with the variable SOCIAL FORM (monogyne or polygyne) replacing the variable GENOTYPE.

Analysis of variance calculations were performed in R. For the genotype comparison, 4,005 clones were removed from the ANOVA analysis because there were not enough data points for the *F*-statistic calculations (for example, for a given clone all the batch_2004 samples for GENOTYPE *BB* had negative intensities and/or were flagged). However, because many genes are represented by multiple independent clones, 95.5% (9,288/9,722) of the putative genes on the microarray were present in the 14,433 clones used in the analyses. Similarly, for the social form analysis 3,791 clones were removed from the ANOVA analysis, with the remaining 14,647 clones representing 96.9% (9,419/9,722) of the putative genes.

We restricted our analyses to the 73 and 139 cDNA clones that satisfied a false discovery rate (FDR) of 10% for the genotype and social form comparisons, respectively [67]. Duplicated clones on the microarray, independent cDNA clones representing the same gene, and sequences with greater than 95% sequence identity were merged and averaged, resulting in 39 genes with significantly different expression in the genotype comparison and 91 in the social form comparison. Expression levels presented in the figures are modified from the loess-normalized log2 expression ratios. For each gene, the batch effect (derived from the ANOVA calculations) was first subtracted from the loess-normalized log2 expression ratio. Then, the batch-adjusted expression ratios were normalized to the average across all experiments (including the two *bb* hybridizations).

Statistical significance of the expression differences detected by the ANOVA calculations was additionally evaluated by means of non-parametric Mann-Whitney tests conducted on the normalized, batch-adjusted data (Figure S1). Expression differences between polygyne workers of different *Gp-9* genotype (*BB*, *Bb*) were confirmed to be highly significant for all 39 genes identified by the ANOVA (all *P*<0.002), as were expression differences between *BB* workers of different social form for all 91 genes identified by the ANOVA (all *P*<0.002). In contrast, among the 39 genes influenced by *Gp-9* genotype, only seven (18%) showed significantly different expression between monogyne and polygyne workers with the *BB* genotype (0.001<*P*<0.041), while among the 91 genes influenced by social form, only three (3.3%) showed significantly different expression between *BB* and *Bb* workers of the polygyne form (0.0001<*P*<0.047) (see Figure S1). Given the large number of these tests performed, some 5% of the significant results are presumed to represent Type I errors.

Expression data were hierarchically clustered and examined using Cluster and Treeview [68]. We also performed SOM (self-organizing map) clustering of the experimental samples (by array) for both the genotype and social form comparisons (data not shown). For the genotype comparisons, the samples clustered into two distinct groups according to genotype (*BB* and *Bb*) and no additional group was uncovered. Similarly, for the social form comparison, all the monogyne samples clustered together while the polygyne samples separated into two groups, those with high and those with low levels of viral gene expression. Because this analysis did not reveal any striking new patterns, the results are not presented in detail.

### Annotation of Differentially Regulated Genes

Because previous annotations of the genes represented on the fire ant microarray [30] may be outdated, we performed new similarity searches against the non-redundant protein sequence database using the BLASTX algorithm [69],[70]. All comparisons were performed on the Blast Network Service provided by the Swiss Institute for Bioinformatics (release July 17, 2007). The default settings were used with an *E*-value threshold of 1e-5, except where otherwise indicated. The accession number of the best match for each gene is reported in Tables 1 and 2, except when it was an *Apis mellifera* gene derived from the Ensembl automatic annotation. In this case, we chose the next best hit, because little is known about gene function in *A. mellifera*, and the genome of this species has been removed from the current Ensembl releases. All BLASTX matches with *E*≤1 (but limited to the top 20) are listed in Tables S3 and S4. Each fire ant gene was also manually assigned to a descriptive category (Tables 1 and 2 and Text S1). The category putatively encapsulates the general function of each gene and is subjectively derived from examining the SwissProt or Ensembl database entries of the five best hits (all *E*<1e-5), with an emphasis on Gene Ontology, Interpro, and PANTHER annotations. To determine which categories were overrepresented in each set of differentially expressed genes, we used a one-tailed hypergeometric test implemented in R [71],[72].

Gene expression data meet Minimum Information About a Microarray Experiment (MIAME) standards and have been deposited at Gene Expression Omnibus (http://www.ncbi.nlm.nih.gov/geo/) with accession number GSE11694.

## Supporting Information

Figure S1 Results of Mann-Whitney statistical tests for gene expression differences.
(0.03 MB PDF)

Table S1 Comparison of qRT-PCR and microarray expression ratios.
(0.02 MB PDF)

Table S2 List of gene categories significantly overrepresented among differentially expressed genes in the genotype and social form comparisons.
(0.02 MB PDF)

Table S3 BLASTX matches for genes differentially expressed between polygyne *Gp-9 BB* and *Gp-9 Bb* workers.
(0.10 MB XLS)

Table S4 BLASTX matches for genes differentially expressed between monogyne and polygyne *Gp-9 BB* workers.
(0.18 MB XLS)

Table S5 Genes, primer sequences, and primer concentrations used for qRT-PCR verification of microarray expression data.
(0.02 MB PDF)

Text S1 Supplementary notes and methods.
(0.07 MB PDF)

## References

[1] Hansen TF (2006). The evolution of genetic architecture.. Annu Rev Ecol Evol Syst.

[2] Greenspan RJ (2004). E pluribus unum, ex uno plura: quantitative and single-gene perspectives on the study of behavior.. Annu Rev Neurosci.

[3] Orr HA (2005). The genetic theory of adaptation: a brief history.. Nat Rev Genet.

[4] Orr HA, Coyne JA (1992). The genetics of adaptation - a reassessment.. Am Nat.

[5] Sokolowski MB (2001). *Drosophila*: genetics meets behaviour.. Nat Rev Genet.

[6] Wolf JB, Brodie ED, Cheverud JM, Moore AJ, Wade MJ (1998). Evolutionary consequences of indirect genetic effects.. Trends Ecol Evol.

[7] Wolf JB, Brodie ED, Moore AJ (1999). Interacting phenotypes and the evolutionary process. II. Selection resulting from social interactions.. Am Nat.

[8] Robinson GE, Grozinger CM, Whitfield CW (2005). Sociogenomics: social life in molecular terms.. Nat Rev Genet.

[9] Linksvayer TA, Wade MJ (2005). The evolutionary origin and elaboration of sociality in the aculeate hymenoptera: Maternal effects, sib-social effects, and heterochrony.. Q Rev Biol.

[10] Linksvayer TA (2006). Direct, maternal, and sibsocial genetic effects on individual and colony traits in an ant.. Evolution.

[11] Nedelcu AM, Michod RE (2006). The evolutionary origin of an altruistic gene.. Mol Biol Evol.

[12] Moore AJ, Brodie ED, Wolf JB (1997). Interacting phenotypes and the evolutionary process. I. Direct and indirect genetic effects of social interactions.. Evolution.

[13] Gardner A, West SA, Barton NH (2007). The relation between multilocus population genetics and social evolution theory.. Am Nat.

[14] Krieger MJB, Ross KG (2002). Identification of a major gene regulating complex social behavior.. Science.

[15] Keller L, Ross KG (1999). Major gene effects on phenotype and fitness: the relative roles of *Pgm-3* and *Gp-9* in introduced populations of the fire ant *Solenopsis invicta*.. J Evol Biol.

[16] Keller L, Ross KG (1993). Phenotypic Basis of Reproductive Success in a Social Insect - Genetic and Social Determinants.. Science.

[17] Keller L, Ross KG (1993). Phenotypic Plasticity and Cultural Transmission of Alternative Social Organizations in the Fire Ant *Solenopsis invicta*.. Behav Ecol Sociolbiol.

[18] Keller L, Ross KG (1995). Gene by Environment Interaction - Effects of a Single-Gene and Social-Environment on Reproductive Phenotypes of Fire Ant Queens.. Functional Ecology.

[19] DeHeer CJ, Goodisman MAD, Ross KG (1999). Queen dispersal strategies in the multiple-queen form of the fire ant *Solenopsis invicta*.. Am Nat.

[20] Keller L, Ross KG (1998). Selfish genes: a green beard in the red fire ant.. Nature.

[21] Ross KG, Keller L (1998). Genetic control of social organization in an ant.. Proc Natl Acad Sci USA.

[22] Gotzek D, Ross KG (2007). Genetic regulation of colony social organization in fire ants: an integrative overview.. Q Rev Biol.

[23] Ross KG, Keller L (2002). Experimental conversion of colony social organization by manipulation of worker genotype composition in fire ants (*Solenopsis invicta*).. Behav Ecol Sociolbiol.

[24] Hallar BL, Krieger MJB, Ross KG (2007). Potential cause of lethality of an allele implicated in social evolution in fire ants.. Genetica.

[25] Tschinkel WR (2006). The fire ants.

[26] Ross KG, Keller L (1995). Ecology and Evolution of social-organization - Insights from fire ants and other highly eusocial insects.. Annu Rev Ecol Syst.

[27] Shoemaker DD, Ross KG (1996). Effects of social organization on gene flow in the fire ant *Solenopsis invicta*.. Nature.

[28] Ross KG, Shoemaker DD (1993). An unusual pattern of gene flow between the 2 social forms of the fire ant *Solenopsis invicta.*. Evolution.

[29] Goodisman MAD, Ross KG, Asmussen MA (2000). A formal assessment of gene flow and selection in the fire ant *Solenopsis invicta*.. Evolution.

[30] Wang J, Jemielity S, Uva P, Wurm Y, Graff J (2007). An annotated cDNA library and microarray for large-scale gene-expression studies in the ant *Solenopsis invicta*.. Genome Biol.

[31] McGraw LA, Gibson G, Clark AG, Wolfner MF (2004). Genes Regulated by Mating, Sperm, or Seminal Proteins in Mated Female *Drosophila melanogaster*.. Curr Biol.

[32] Harbison ST, Chang S, Kamdar KP, Mackay TFC (2005). Quantitative genomics of starvation stress resistance in *Drosophila*.. Genome Biol.

[33] Morozova TV, Anholt RRH, Mackay TFC (2006). Transcriptional response to alcohol exposure in *Drosophila melanogaster*.. Genome Biol.

[34] Andronopoulou E, Labropoulou V, Douris V, Woods DF, Biessmann H (2006). Specific interactions among odorant-binding proteins of the African malaria vector *Anopheles gambiae*.. Insect Mol Biol.

[35] Wang P, Lyman RF, Shabalina SA, Mackay TFC, Anholt RRH (2007). Association of polymorphisms in odorant-binding protein genes with variation in olfactory response to benzaldehyde in *Drosophila*.. Genetics.

[36] Mescher MC (2001). Levels of selection and the evolution of social organization [PhD dissertation]: University of Georgia. vi, 104 leaves..

[37] Rice WR (1987). Genetic hitchhiking and the evolution of reduced genetic activity of the Y sex chromosome.. Genetics.

[38] Bachtrog D (2006). A dynamic view of sex chromosome evolution.. Curr Opin Genet Dev.

[39] Charlesworth D, Charlesworth B, Marais G (2005). Steps in the evolution of heteromorphic sex chromosomes.. Heredity.

[40] Ross KG (1997). Multilocus evolution in fire ants: effects of selection, gene flow and recombination.. Genetics.

[41] Krieger MJB, Ross KG (2005). Molecular evolutionary analyses of the odorant-binding protein gene *Gp-9* in fire ants and other *Solenopsis* species.. Mol Biol Evol.

[42] Macom TE, Porter SD (1996). Comparison of polygyne and monogyne red imported fire ant (Hymenoptera: Formicidae) population densities.. Ann Entomol Soc Am.

[43] Oi DH, Valles SM, Pereira RM (2004). Prevalence of *Thelohania solenopsae* (Microsporidia : Thelohaniidae) infection in monogyne and polygyne red imported fire ants (Hymenoptera : Formicidae).. Environ Entomol.

[44] Valles SM, Strong CA, Hashimoto Y (2007). A new positive-strand RNA virus with unique genome characteristics from the red imported fire ant, *Solenopsis invicta*.. Virology.

[45] Raj PA, Dentino AR (2002). Current status of defensins and their role in innate and adaptive immunity.. FEMS Microbiol Lett.

[46] Goodisman MAD, Sankovich KA, Kovacs JL (2007). Genetic and morphological variation over space and time in the invasive fire ant *Solenopsis invicta*.. Biol Invasions.

[47] Goodisman MAD, Mack PD, Pearse DE, Ross KG (1999). Effects of a single gene on worker and male body mass in the fire ant *Solenopsis invicta* (Hymenoptera : Formicidae).. Ann Entomol Soc Am.

[48] Edwards AC, Rollmann SM, Morgan TJ, Mackay TF (2006). Quantitative genomics of aggressive behavior in *Drosophila melanogaster*.. PLoS Genet.

[49] Roberge C, Einum S, Guderley H, Bernatchez L (2006). Rapid parallel evolutionary changes of gene transcription profiles in farmed Atlantic salmon.. Mol Ecol.

[50] Jarvis ED, Scharff C, Grossman MR, Ramos JA, Nottebohm F (1998). For whom the bird sings: Context-dependent gene expression.. Neuron.

[51] Whitfield CW, Cziko AM, Robinson GE (2003). Gene expression profiles in the brain predict behavior in individual honey bees.. Science.

[52] Burmeister SS, Jarvis ED, Fernald RD (2005). Rapid behavioral and genomic responses to social opportunity.. PLoS Biol.

[53] Cummings ME, Larkins-Ford J, Reilly CR, Wong RY, Ramsey M (2008). Sexual and social stimuli elicit rapid and contrasting genomic responses.. Proc Biol Sci.

[54] Linksvayer TA (2007). Ant Species Differences Determined by Epistasis between Brood and Worker Genomes.. PLoS ONE.

[55] Agrawal AF, Brodie ED, Brown J (2001). Parent-offspring coadaptation and the dual genetic control of maternal care.. Science.

[56] Hunt J, Simmons LW (2002). The genetics of maternal care: Direct and indirect genetic effects on phenotype in the dung beetle *Onthophagus taurus*.. Proc Natl Acad Sci USA.

[57] Rauter CM, Moore AJ (2002). Evolutionary importance of parental care performance, food resources, and direct and indirect genetic effects in a burying beetle.. J Evol Biol.

[58] Amdam GV, Csondes A, Fondrk MK, Page RE (2006). Complex social behaviour derived from maternal reproductive traits.. Nature.

[59] Grozinger CM, Fan Y, Hoover SE, Winston ML (2007). Genome-wide analysis reveals differences in brain gene expression patterns associated with caste and reproductive status in honey bees (*Apis mellifera*).. Mol Ecol.

[60] Toth AL, Varala K, Newman TC, Miguez FE, Hutchison SK (2007). Wasp gene expression supports an evolutionary link between maternal behavior and eusociality.. Science.

[61] Dawkins R (1999). The extended phenotype: the long reach of the gene.

[62] Jouvenaz DP, Allen GE, Banks WA, Wojcik DP (1977). Survey for Pathogens of Fire Ants, *Solenopsis*-Spp-(Hymenoptera-Formicidae) in Southeastern United-States.. Fla Entomol.

[63] Shoemaker DD, DeHeer CJ, Krieger MJB, Ross KG (2006). Population genetics of the invasive fire ant *Solenopsis invicta* (Hymenoptera : Formicidae) in the United States.. Ann Entomol Soc Am.

[64] http://derisilab.ucsf.edu/

[65] Randolph JB, Waggoner AS (1997). Stability, specificity and fluorescence brightness of multiply-labeled fluorescent DNA probes.. Nucleic Acids Res.

[66] Gentleman RC, Carey VJ, Bates DM, Bolstad B, Dettling M (2004). Bioconductor: open software development for computational biology and bioinformatics.. Genome Biol.

[67] Benjamini Y, Hochberg Y (1995). Controlling the False Discovery Rate - a Practical and Powerful Approach to Multiple Testing.. J R Stat Soc Ser B.

[68] Eisen MB, Spellman PT, Brown PO, Botstein D (1998). Cluster analysis and display of genome-wide expression patterns.. Proc Natl Acad Sci U S A.

[69] Altschul SF, Gish W, Miller W, Myers EW, Lipman DJ (1990). Basic local alignment search tool.. J Mol Biol.

[70] Altschul SF, Madden TL, Schaffer AA, Zhang J, Zhang Z (1997). Gapped BLAST and PSI-BLAST: a new generation of protein database search programs.. Nucleic Acids Res.

[71] Ihaka R, Gentleman R (1996). R: A language for data analysis and graphics.. J Comp Graph Stat.

[72] Rivals I, Personnaz L, Taing L, Potier MC (2007). Enrichment or depletion of a GO category within a class of genes: which test?. Bioinformatics.

