# Supplementary figures and images for: Genome-Wide Expression Patterns and the Genetic Architecture of a Fundamental Social Trait
Source: PLoS Genet. 2008 Jul 18;4(7):e1000127. doi: 10.1371/journal.pgen.1000127 (PMC2442221; doi:10.1371/journal.pgen.1000127)

# Figure S1

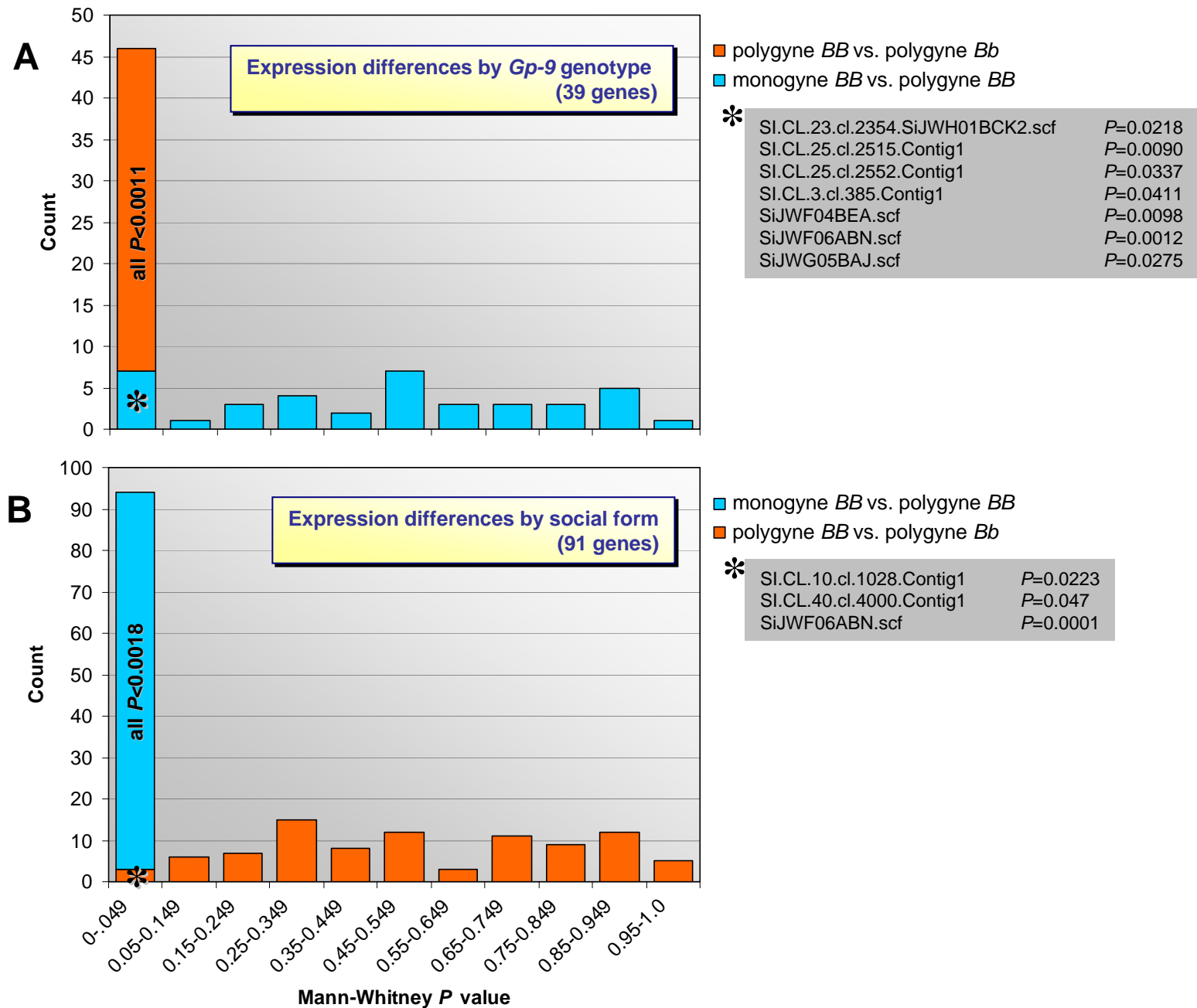

Supplement: Figure S1 — Results of Mann-Whitney statistical tests for gene expression differences. (0.03 MB PDF) [file pgen.1000127.s001.pdf]
