# Supplementary material for: Genome-Wide Expression Patterns and the Genetic Architecture of a Fundamental Social Trait
Source: PLoS Genet. 2008 Jul 18;4(7):e1000127. doi: 10.1371/journal.pgen.1000127 (PMC2442221; doi:10.1371/journal.pgen.1000127)
Supplement: Table S1 — Comparison of qRT-PCR and microarray expression ratios. (0.02 MB PDF) [file pgen.1000127.s002.pdf]

Supplementary Table S1. Comparison of qRT-PCR and microarray expression ratios.

Microarray data are given for the 20 colonies studied in the main experiments as well as the subset of ten colonies used for the qRT-PCR analyses.

Gene product	qRT-PCR (10 colonies)		microarray (10 colonies)		microarray (20 colonies)	
Gp-9 genotype comparison	expression ratio *Bb/BB*	*P*-value*	expression ratio *Bb/BB*	*P*-value*	expression ratio *Bb/BB*	*P*-value*
Ankyrin repeat and FYVE domain containing protein	1.58	<0.001	1.22	0.002	1.20	<0.001
piggyBac transposon	15108.77	<0.001	33.76	<0.001	28.94	<0.001
odorant binding protein #1	0.53	0.008	0.41	0.002	0.49	<0.001
odorant binding protein #2	1.56	0.008	1.54	0.005	1.69	<0.001
Social form comparison	expression ratio P/M	*P*-value*	expression ratio P/M	*P*-value*	expression ratio P/M	*P*-value*
alpha-glucosidase (hbg3)	0.91	0.362	0.80	0.011	0.78	<0.001
defensin-2	0.75	0.038	0.70	0.002	0.70	<0.001
prefoldin subunit 4	1.22	0.064	1.10	0.027	1.17	<0.001
nonstructural protein of *S. invicta* virus 2	441.34	<0.001	112.10	<0.001	87.39	<0.001

**P*-values for differences in expression from 1-tailed Student's *t*-tests.
