# Supplementary material for: Genome-Wide Expression Patterns and the Genetic Architecture of a Fundamental Social Trait
Source: PLoS Genet. 2008 Jul 18;4(7):e1000127. doi: 10.1371/journal.pgen.1000127 (PMC2442221; doi:10.1371/journal.pgen.1000127)
Supplement: Table S2 — List of gene categories significantly overrepresented among differentially expressed genes in the genotype and social form comparisons. (0.02 MB PDF) [file pgen.1000127.s003.pdf]

Supplementary Table S2. List of gene categories significantly overrepresented among differentially expressed genes in the genotype and social form comparisons

Category†	Genotype comparison		Social form comparison		Genes on array
	Number of genes	Fold enrichment	Number of genes	Fold enrichment	
allergen	2	**35.8** $p < 0.0014$	1		17
mitochondrial	1		8	**2.3** $p < 0.022$	450
nucleic acid metabolism	1		2	0.4 $p < 0.98$	737
odorant binding	3	**38.0** $p < 6.4e-5$	0		24
prefoldin complex	0		3	**65.2** $p < 8.6e-6$	6
transferase	0		2	0.6	444
transposon	2	**6.1** $p < 0.042$	0		99
virus	0		6	**27.9** $p < 5.7e-8$	28
TOTAL	39		91		11864

Gene groups with 0 or 1 gene were not analyzed.
† Gene category as described in the supplementary text S1.
