# Supplementary material for: Genome-Wide Expression Patterns and the Genetic Architecture of a Fundamental Social Trait
Source: PLoS Genet. 2008 Jul 18;4(7):e1000127. doi: 10.1371/journal.pgen.1000127 (PMC2442221; doi:10.1371/journal.pgen.1000127)
Supplement: Table S5 — Genes, primer sequences, and primer concentrations used for qRT-PCR verification of microarray expression data. (0.02 MB PDF) [file pgen.1000127.s006.pdf]

Supplementary Table S5. Genes, primer sequences, and primer concentrations used for qRT-PCR verification of microarray expression data

Fire ant gene	Gene product	Forward primer	Reverse primer	Primer concentration
SI.CL.7.cl.734.Contig1	Elongation factor 1 alpha	CAGCCGATGTAGCTCACCCT	TGTCAAATTCGTCTCCCGTG	300 nM
SI.CL.19.cl.1975.Contig1	Ribosomal protein S9	GTGACGATCTTTCGGCATGG	TTGGAGAAGACCGACGGAAT	200 nM
SI.CL.40.cl.4088.Contig1	Ankyrin repeat and FYVE domain containing protein	CATCACTGCCGACATTGTGG	TTGACCAGAACACTTGCTGCA	300 nM
SI.CL.18.cl.1888.Contig1	odorant binding protein #1	AACTCCTGCTGACTTTGTCAAACC	CACGCTATAAAGCAGCCATGTT	300 nM
SI.CL.3.cl.385.Contig1	odorant binding protein #2	GTGGGTCCTACCAGGTGAATTTG	GTCATGTTTGCTTTTGCGAGAT	300 nM
SiJWF04BEA.scf	piggyBac transposon	GTGCACAACATCAACTTGGTT	TTGCACAATACGGATCTTCG	300 nM
SI.CL.20.cl.2059.Contig1	defensin-2	CCTTCTGTCCTGGCAGTCCA	GCGCAAGCACTGTGATTGAT	150 nM
SI.CL.11.cl.1163.Contig1	alpha-glucosidase (hbg3)	CAAAAAGAACGGGACGACTCC	GCCATTTTCGCAGAGCAGA	300 nM
SI.CL.21.cl.2171.Contig1	prefoldin subunit 4	AGGCAAAATGCAAAATTGGAA	CGGAAGCATCCTCCAAATTC	300 nM
SI.CL.6.cl.610.Contig1	non-structural protein of *S. invicta* virus 2	ATTGCATCGACCTGTTGTGTG	TGAAGTCTGTGCGGAGGTTCT	150 nM
