# Supplementary material for: Genome-Wide Expression Patterns and the Genetic Architecture of a Fundamental Social Trait
Source: PLoS Genet. 2008 Jul 18;4(7):e1000127. doi: 10.1371/journal.pgen.1000127 (PMC2442221; doi:10.1371/journal.pgen.1000127)
Supplement: Text S1 — Supplementary notes and methods. (0.07 MB PDF) [file pgen.1000127.s007.pdf]

SUPPLEMENTARY TEXT S1 – Supplementary notes and methods

I) Verification of gene expression patterns by quantitative real-time RT-PCR (qRT-PCR)

We used qRT-PCR to verify the expression patterns of four genes with expression levels influenced by focal worker *Gp-9* genotype and another four genes with expression levels influenced by the social environment. Six of the eight genes were chosen because their products potentially directly influence traits related to colony social organization (see main text) and because they display a range of magnitudes of differential expression (1.17-fold to 87.3-fold differences). The remaining two were chosen because they exhibited little differential gene expression in our experiments (1.20-fold and 1.28-fold differences) but displayed interesting expression patterns in other (unpublished) microarray experiments. All analyses were conducted using RNA samples from the ten colonies of each social form for which sufficient RNA remained following the microarray experiments. Thus, we obtained qRT-PCR expression profiles for *BB* and *Bb* workers from ten polygyne colonies and *BB* workers from ten monogyne colonies.

We generated cDNA by reverse transcription of amplified RNA as follows. Amplified RNA (1 µg) was combined with 1.25 µL of random hexamers (0.2 µg/µL), 1 µL of dNTPs (10 µM), and water to a final volume of 12 µL. This mixture was heated at 65°C for 5 min and then placed on ice for 5 min. Next, a master mix containing 4 µL of 5x First-strand buffer, 2 µL DTT (0.1 M), 1 µL RNase Inhibitor (Invitrogen), and 1 µL of reverse transcriptase enzyme (Superscript III, Invitrogen) was added, and the resulting mixture was incubated at 25°C for 10 min. Reverse transcription was performed at 50°C for 1 h. A 40-fold dilution of the resulting cDNA was used for the quantitative PCR.

The qRT-PCR amplification mixtures were made by combining 2 µL of diluted cDNA with 5 µL of Power SYBR Green PCR Master Mix (Applied Biosystems) and the appropriate gene-specific concentrations of each forward and reverse primer (Table S5) to make up a final volume of 10 µL. PrimerExpress software (ABI) was used to design specific primers (Table S5). Reactions were run in triplicate on an ABI PRISM 7900HT Sequence Detector (Applied Biosystems) using the default parameters defined by the manufacturer.

Results from Sequence Detection Systems software (Applied Biosystems; fluorescence intensity default threshold set at 0.2) were exported as tab-delimited files into qBase [1] for subsequent analysis. Thirty amplified RNA samples (ten each for monogyne *BB*, polygyne *BB*, and polygyne *Bb* samples) were analyzed for each gene, including the two control genes. Data were filtered for outliers (max dCt = 0.5) and relative gene expression levels were calculated with PCR efficiency correction and reference gene normalization to the control genes. The genes *EF1-alpha* and *Rps9* were selected as controls because they exhibited no differential expression in this and other (unpublished) microarray and qRT-PCR studies. Statistical analyses of relative gene expression levels were performed in Microsoft Excel and R.

The qRT-PCR and microarray expression results matched well. Differences in expression levels were in the same direction for all eight genes (Table S1). Moreover, the gene expression ratios were highly correlated between the qRT-PCR and microarray experiments ($P=0.0012$, Spearman's rank correlation test). Finally, the differences in gene expression revealed by qRT-PCR were statistically significant for six genes ($P<0.05$) and marginally significant for a seventh ($P=0.064$) despite the sample sizes being only half those used in the microarray experiments (Table S1).

II) Gene category analysis

To determine whether particular gene categories were overrepresented among the genes differentially expressed in the genotype and social form comparisons, we used the cumulative hypergeometric probability. The fold enrichment was calculated as the proportion of genes of a particular category found in the experiment divided by the proportion expected

by chance. The proportion expected by chance is equivalent to the total number of genes in a gene class divided by all the genes on the microarray.

We determined the total number of genes in each group for all gene categories containing ≥ 2 genes differentially expressed in either the genotype or social form comparisons. A gene was counted if it contained the appropriate Gene Ontology (GO) term, possessed the right keywords in the one line descriptions for the best hit, or was significantly similar to well-annotated genes within the category by BLAST analysis.

Numbers of genes in each gene category:

Allergen (17 genes).
We identified 12 fire ant genes with the word "allergen" in the one line description for the best hit. We identified an additional five putative fire ant allergen genes by comparing the major allergen of cockroach (Q9UAM5) against the fire ant genes using TBLASTN.

Prefoldin chaperone (6 genes).
There are six prefoldin subunit genes in metazoan genomes. All six are present on the microarray.

Mitochondrial (estimated 450 genes).
In the fire ant cDNA library, 300 genes are annotated with the GO term "mitochondrion". However, only 6 out of 9 mitochondrial genes, determined by manual examination of all the 129 differentially expressed genes, were electronically annotated with the GO term "mitochondrion". Using this success rate (67%) for the identification of mitochondrial genes, we estimate the microarray to contain approximately 450 such genes.

Nucleic acid metabolism (737 genes).
In the fire ant cDNA library, 737 genes are annotated with the GO term "nucleobase, nucleoside, nucleotide and nucleic acid metabolism".

Odorant binding (24 genes)
In the fire ant cDNA library, 19 genes are annotated with the GO term "odorant binding". Odorant binding proteins include chemosensory proteins, and five additional chemosensory proteins were identified by the one line description for the best hit and Interpro annotation.

Transferase (444 genes)
In the fire ant cDNA library, 444 genes are annotated with the GO term "transferase activity".

Transposon (99 genes)
In the fire ant cDNA library, 61 genes are annotated with the GO term "transposase" or "RNA-directed DNA polymerase activity". An additional 38 transposons were identified by the one line description for the best hit.

Virus (28 genes)
In the fire ant cDNA library, 15 genes are annotated with the GO term "viral life cycle" or "virion". Eight viral genes were identified by the one line description for the best hit. An additional five viral genes were identified to encode portions of the fire ant virus, SINV-2 genome.

REFERENCES

1. Hellemans J, Mortier G, De Paepe A, Speleman F, Vandesompele J (2007) qBase relative quantification framework and software for management and automated analysis of real-time quantitative PCR data. Genome Biology 8.
